# Supplementary material for: Teledentistry for improving access to, and quality of oral health care: A protocol for an overview of systematic reviews and meta-analyses
Source: PLoS One. 2024 Jan 2;19(1):e0288677. doi: 10.1371/journal.pone.0288677 (PMC10760664; doi:10.1371/journal.pone.0288677)
Supplement: S1 File — (DOCX) [file pone.0288677.s002.docx]

**Search strategy**

**Medline (OVID)**

**Date of the search:** 21-06-2023

**Database limit:** No database limit has been applied.

| **#** | **Search strategy** | **Results** |
| --- | --- | --- |
| 1 | Teledentistry.ti,ab,kf,kw OR "tele-dentistry".ti,ab,kf,kw |  |
| 2 | Dental Health Services/ OR Dentists/ OR exp Dentistry/ OR exp Mouth Neoplasms/ |  |
| 3 | dental.ti,ab,kf,kw OR dentist*.ti,ab,kf,kw OR orthodonti*.ti,ab,kf,kw OR periodont*.ti,ab,kf,kw  OR prosthodont*.ti,ab,kf,kw OR (oral adj2 (health OR care OR surger* OR Diagnos* OR Hygiene OR Medicine OR lesion?)).ti,ab,kf,kw OR ((oral OR Mouth) adj1 (cancer OR Neoplasm?)).ti,ab,kf,kw |  |
| 4 | 2 OR 3 |  |
| 5 | exp Telemedicine/ OR Mobile Applications/ OR exp Cell Phone/ OR exp Videoconferencing/ |  |
| 6 | Telemedicine.ti,ab,kf,kw OR Telehealth.ti,ab,kf,kw OR Teleconsultation.ti,ab,kf,kw OR telediagnosis.ti,ab,kf,kw OR teletriage.ti,ab,kf,kw OR telemonitoring.ti,ab,kf,kw OR mHealth.ti,ab,kf,kw OR eHealth.ti,ab,kf,kw OR "e-health".ti,ab,kf,kw OR "Mobile Health".ti,ab,kf,kw OR (remote adj2 (telecommunication OR Consultation OR care OR diagnosis)).ti,ab,kf,kw OR (tele adj2 (medicine OR consultation* OR health OR diagnosis OR triage)).ti,ab,kf,kw OR (virtual adj2 (consult* OR care)).ti,ab,kf,kw OR econsult*.ti,ab,kf,kw  OR "e consult*".ti,ab,kf,kw OR ((Video OR Electronic) adj2 Consult*).ti,ab,kf,kw OR "mobile app*".ti,ab,kf,kw OR "cellular phone".ti,ab,kf,kw OR smartphone*.ti,ab,kf,kw OR "text messag*".ti,ab,kf,kw OR "mobile phone".ti,ab,kf,kw OR Videoconferenc*.ti,ab,kf,kw |  |
| 7 | 5 OR 6 |  |
| 8 | Meta-Analysis/ OR Systematic Review/ OR Review/ |  |
| 9 | (scoping adj2 (stud* OR review?)).ti,ab,kf,kw OR (evidence adj2 map*).ti,ab,kf,kw OR "Environmental scan*".ti,ab,kf,kw OR "evidence synthesis".ti,ab,kf,kw OR "systematic map*".ti,ab,kf,kw OR overview?.ti,ab,kf,kw OR "review of review?".ti,ab,kf,kw OR "rapid review?".ti,ab,kf,kw OR (Narrative adj1 (review OR summary OR Synthesis)).ti,ab,kf,kw OR (mixed adj1 (method? OR stud*)).ti,ab,kf,kw OR "Research Synthesis".ti,ab,kf,kw OR "Meta-synthesis".ti,ab,kf,kw OR "Meta-Theory".ti,ab,kf,kw OR "Meta-Study".ti,ab,kf,kw OR "Meta Ethnography".ti,ab,kf,kw OR "Meta-aggregation".ti,ab,kf,kw OR "Meta-narrative".ti,ab,kf,kw OR "Meta-interpretation".ti,ab,kf,kw OR "Meta-Analysis".ti,ab,kf,kw OR "Umbrella review?".ti,ab,kf,kw OR "systematic review?".ti,ab,kf,kw OR "mapping review?".ti,ab,kf,kw OR (Integrative OR Realist) adj2 (Review? OR Synthesis).ti,ab,kf,kw OR ((comprehensive OR literature OR "State-of-the-art") adj1 Review?).ti,ab,kf,kw |  |
| 10 | 8 OR 9 |  |
| 11 | 4 AND 7 |  |
| 12 | (1 OR 11) AND 10 | 198 |
