# Supplementary material for: Teledentistry for improving access to, and quality of oral health care: A protocol for an overview of systematic reviews and meta-analyses
Source: PLoS One. 2024 Jan 2;19(1):e0288677. doi: 10.1371/journal.pone.0288677 (PMC10760664; doi:10.1371/journal.pone.0288677)
Supplement: S2 File — (DOCX) [file pone.0288677.s003.docx]

**Data extraction sheet**

| **Title of the review:** **Teledentistry for improving access to, and quality of oral health care: A protocol for an overview of systematic reviews and meta-analyses** | |
| --- | --- |
| **Reviewer’s name** |  |
| **Publication details** | Title |
|  | Year of publication |
|  | First author |
|  | Corresponding author |
|  | Country in which the study is conducted |
|  | Language of publication |
|  |  |
| **Study details** | Objectives |
|  | Sources searched |
|  | Inclusion criteria |
|  | Exclusion criteria |
|  | Intervention |
|  | Comparators |
|  | Setting |
|  |  |
| **Methodological information** | Type of study (Quantitative, qualitative or mixed) |
|  | Theoretical approach (Y, N, NR) |
|  | Name of theoretical approach if yes |
|  | Study design of the primary studies |
|  | Domains in dentistry |
|  |  |
|  |  |
| **Participant information** | Participant profile (OHCP) |
|  | Type of speciality if reported: e.g. orthodontics, periodontics, prosthodontics, endodontics, pediatrics, OMFS/Oral medicine or other |
|  | Age (mean, SD) of the oral health care providers |
|  | Participants (types of patients) |
|  | Age (mean, SD) of the patients |
|  | Other participants |
| **Characteristics of teledentistry** | Types of teledentistry modalities e.g. synchronous, asynchronous, remote monitoring, mhealth |
|  | Technology used e.g. adobe connect, skype, whatsapp, mobile phone, email, facebook |
|  |  |
| **Findings** | Primary outcomes |
|  | Secondary outcomes |
|  | Type of data analysis |
|  | Meta analysis (Y/N) |
|  | Main findings |
|  | Results of the meta analysis |
|  | Heterogeneity |
|  | Certainty of evidence |
|  |  |
| **Quality assessment** | Appraisal tools used |
|  | Appraisal rating |
|  |  |
| **Others** | Limitations |
|  | Funding |
|  | Conflict of interest |
|  | Areas for future research |
